# The effect of therapeutic physical modalities on pain, function, and quality of life in patients with myofascial pain syndrome: a systematic review

**DOI:** 10.1186/s12891-023-06418-6

**Published:** 2023-05-12

**Authors:** Peijue He, Wenxuan Fu, Hang Shao, Meng Zhang, Zhuoli Xie, Juan Xiao, Lijuan Li, Yiwei Liu, Yi Cheng, Qian Wang

**Affiliations:** 1 Rehabilitation Medicine Center, West China Hospital, Sichuan University, Chengdu, Sichuan People’s Republic of China; 2 Rehabilitation Medicine Key Laboratory of Sichuan Province, Chengdu, Sichuan People’s Republic of China; 3 Department of Clinical Nutrition, West China Hospital, Sichuan University, Chengdu, China

**Keywords:** Myofascial pain syndrome, Therapeutic physical modalities, Systematic review, Transcutaneous electrical nerve stimulation, Laser, Extracorporeal shock wave therapy

## Abstract

**Background:**

Myofascial pain syndrome (MPS) is a common musculoskeletal pain and dysfunction, which is characterised by myofascial trigger points. Therapeutic physical modalities, as potentially effective treatment options, are commonly used in the clinical setting for the patients with MPS.

**Objective:**

This systematic review aimed to evaluate the safety and effectiveness of therapeutic physical modalities in the treatment of MPS, investigate its therapeutic mechanisms and provide a scientific evidence-based decision.

**Methods:**

According to Preferred Reporting Items for Systematic Reviews and Meta-Analyses guidelines, the PubMed, Cochrane Central Library, Embase, and CINAHL databases were searched for randomized controlled clinical studies published from database inception to October 30, 2022. A total of 25 articles met the study inclusion criteria. Data were extracted from these studies and a qualitative analysis was performed.

**Results:**

Transcutaneous electrical nerve stimulation therapy, extracorporeal shock wave therapy, laser therapy, and other therapeutic physical modalities have been demonstrated to improve the pain symptoms, joint mobility, psychological state, and quality of life in the patients with MPS and no side effects have been reported. The curative effect of therapeutic physical modalities was found to be possibly associated with increased blood perfusion and oxygen supply in ischaemic tissues, reduced hyperalgesia in the peripheral and central nerves, and decreased involuntary muscle contractions.

**Conclusion:**

The systematic review has shown that therapeutic physical modalities could provide a safe and effective therapeutic option for MPS. However, the consensus is currently lacking regarding the optimal treatment paradigm, therapeutic parameters, and mutual combination of therapeutic physical modalities. The clinical trials with robust quality are required to further promote the evidence-based application of therapeutic physical modalities for MPS.

## Introduction

Myofascial pain syndrome (MPS) is a regional pain syndrome whose clinical symptoms are mainly characterized by the presence of highly irritating nodules, namely myofascial trigger points (MTrPs), in a taut band of a single muscle or muscle group accompanied by stiffness, fatigue, tenderness, and pain, muscle spasm and contraction, and limited range of joint motion [1–5]. MPS is the most common source of musculoskeletal pain. Approximately 30–50% of patients with musculoskeletal symptoms suffer from MPS, whose incidence is higher in women [3]. The trapezius, rhombus, infraspinatus, levator scapulae, and paravertebral muscles are most commonly involved [6–8]. Patients with chronic MPS are under considerable physical and psychological pressure and tend to have depression or anxiety and impaired quality of life (QOL) [1–5]. A reported 61% of patients with chronic MPS have mild to moderate anxiety, the level of which is related to the baseline pain severity, suggesting a correlation between the two [9].

Research to date on the aetiology of MPS reported that myofascial injury manifests as microtears of myofascial tissue, inflammatory reactions, and muscle fibre contractions that lead to vasoconstriction and circulatory disturbances and reduce the ability to remove metabolic waste, resulting in ischaemia and hypoxia of the muscle tissue and local oedema, forming a spasm-ischaemia-pain cycle [3, 4, 10, 11]. The calcium released by injured muscle combines with ATP, and the abnormal increase in acetylcholine leads to uncontrolled muscle fibre contraction, resulting in muscle fibre bundle tension and shortening, in turn leading to local metabolic activities that result in the release of histamine, bradykinin, 5-hydroxytryptamine, prostaglandins, and other substances that increase sensory nerve fibre sensitivity [3, 12, 13]. Afferent nerves transmit the pain signal to the spinal cord, producing a central pain signal that increases further and expands to the adjacent spinal cord segment, resulting in referred pain [10]. Simultaneously, macrophages and fibroblasts within the muscle fascia are activated and connective tissue proliferates, leading to tissue sclerosis [10, 14]. This series of changes lead to the formation of one or more active MTrPs in the muscles, which in turn leads to MPS pain and dysfunction.

Transcutaneous electrical nerve stimulation (TENS), extracorporeal shock wave therapy (ESWT), and ultrasound (US), are widely used as therapeutic physical modalities in the clinical treatment of MPS. Clinical studies have been conducted to demonstrate the positive role and mechanism of TENS, ESWT, and US in effectively alleviating the MPS symptoms [3, 7, 13, 15–24]. A comprehensive qualitative and quantitative analyses from 9 articles have been performed, demonstrating the positive role of TENS in reducing the pain at the MTrPs within muscle [1]. Similarly, the ESWT has been reported to activate the regeneration process of structural elements of the vertical-motor segment by improving the blood circulation and cell membrane permeability, and ultimately reduce the MPS symptoms [25]. The continuous US had been proved to be superior to the pulsed US in relieving resting pain in the patients with MPS [24].

It has been shown that therapeutic physical modalities possibly relieve pain by increasing blood perfusion and blood oxygen supply in ischaemic tissues, reducing hyperalgesia in the peripheral and central nerves, and decreasing involuntary muscle contractions [2–43]. However, heterogeneous study design and various kinds of therapeutic physical modalities make it difficult to systematically analyse and evaluate the safety, efficacy and protocol of therapeutic physical modalities in the treatment of MPS. Therefore, this study aimed to provide a comprehensive systematic review of therapeutic physical modalities in the treatments of MPS; and to objectively evaluate the safety, efficacy, protocol, parameters, and possible mechanisms for MPS. This review will provide a research foundation for the clinical application of TENS, ESWT, US, transcranial direct-current stimulation (tDCS), laser, and biofeedback in the treatment of MPS.

Therefore, the main research question for this systematic review was as follows: Are therapeutic physical modalities safe and effective for the treatment of pain and dysfunction in patients with MPS? What are the optimal treatment protocol and parameters? and what are the therapeutic mechanisms related to MPS?

## Material and methods

The study protocol was finalised a priori by all authors, and the objectives, electronic search strategy, study inclusion/exclusion criteria, data collection, outcomes of interest, and analytical approaches were defined. This systematic review was conducted in accordance with the Preferred Reporting Items for Systematic Reviews and Meta-Analyses statement.

### Search strategy

A comprehensive bibliographic search was performed of Medline (PubMed), Cochrane Central Library, Embase, and CINAHL for suitable articles published between inception and 30 October 2022. The following medical subject heading terms (MeSH) and free words were used in combination with Boolean operators (AND, OR, NOT): myofascial pain syndrome, electric stimulation therapy, transcutaneous electric nerve stimulation, hydrotherapy, phototherapy, laser therapy, ultraviolet therapy, neurofeedback, transcranial direct current stimulation, magnetic field therapy, ultrasonic therapy, extracorporeal shock wave therapy, traction, and compression. Two independent reviewers screened the titles and abstracts of the articles retrieved in the initial search and then reviewed the full texts of potentially eligible studies. Discrepancies between reviewers were resolved through discussion, and a final decision was reached by consensus with a third reviewer. The reference lists of the included studies were manually searched for additional potentially relevant studies.

### Study types

Randomised controlled trials (RCTs) that investigated the effects of therapeutic physical modalities on MPS were included in this review. Only studies published in English were included in this review. Systematic reviews, crossover trials, case–control studies, cohort studies, and controlled studies were excluded.

### Inclusion and exclusion criteria

All controlled clinical experiments published in English of the therapeutic physical modalities involved in treating MPS were included in this systematic review. The exclusion criteria were as follows: (1) uncontrolled study design; (2) chronic widespread pain, radiculopathy, or other neurological disorders; (3) irrelevant topic, clinical trials, reviews and meta-analyses, editorials, perspectives, and letters to the editor; (4) no outcomes of interest; and (5) animal studies.

### Type of intervention

The intervention was included as follows: (1) ESWT, (2) laser therapy, (3) TENS, (4) ultrasound (US), (5) tDCS, (6) biofeedback, (7) traction, (8) far-infrared ray (FIR), (9) transfer energy capacitive and resistive (TECAR) therapy, and (10) whirlpool bath.

### Outcome measures

Outcome measures included the following: (1) visual analogue scale (VAS) score; (2) pain pressure threshold (PPT); (3) neck range of motion (ROM); (4) Disabilities of the Arm, Shoulder, and Hand score; (5) Neck Disability Index (NDI); (6) Medical Outcomes Study 36-item Short Form health survey (SF-36); (7) Beck Depression Inventory score; (8) Beck Depression Questionnaire score; (9) Pittsburgh Sleep Quality Index; (10) Nottingham Health Profile; (11) 4-item Likert scale score; (12) Neck Functionality Impairment score; (13) QOL; (14) Numerical Rating Scale (NRS) score; (15) surface electromyography (sEMG); (16) Patient Global Impression of Improvement; (17) Functional Assessment of Chronic Illness Therapy; (18) severity of palpable muscle spasm (five-step scale) score; (19) Neck Pain and Disability Scale score; (20) Short Form McGill Pain Questionnaire score; (21) MTrPs activation degree; (22) maximal pain tolerance; (23) lunge test score; and (24) absolute temperature.

## Results

### Study characteristics

**Fig. 1:**
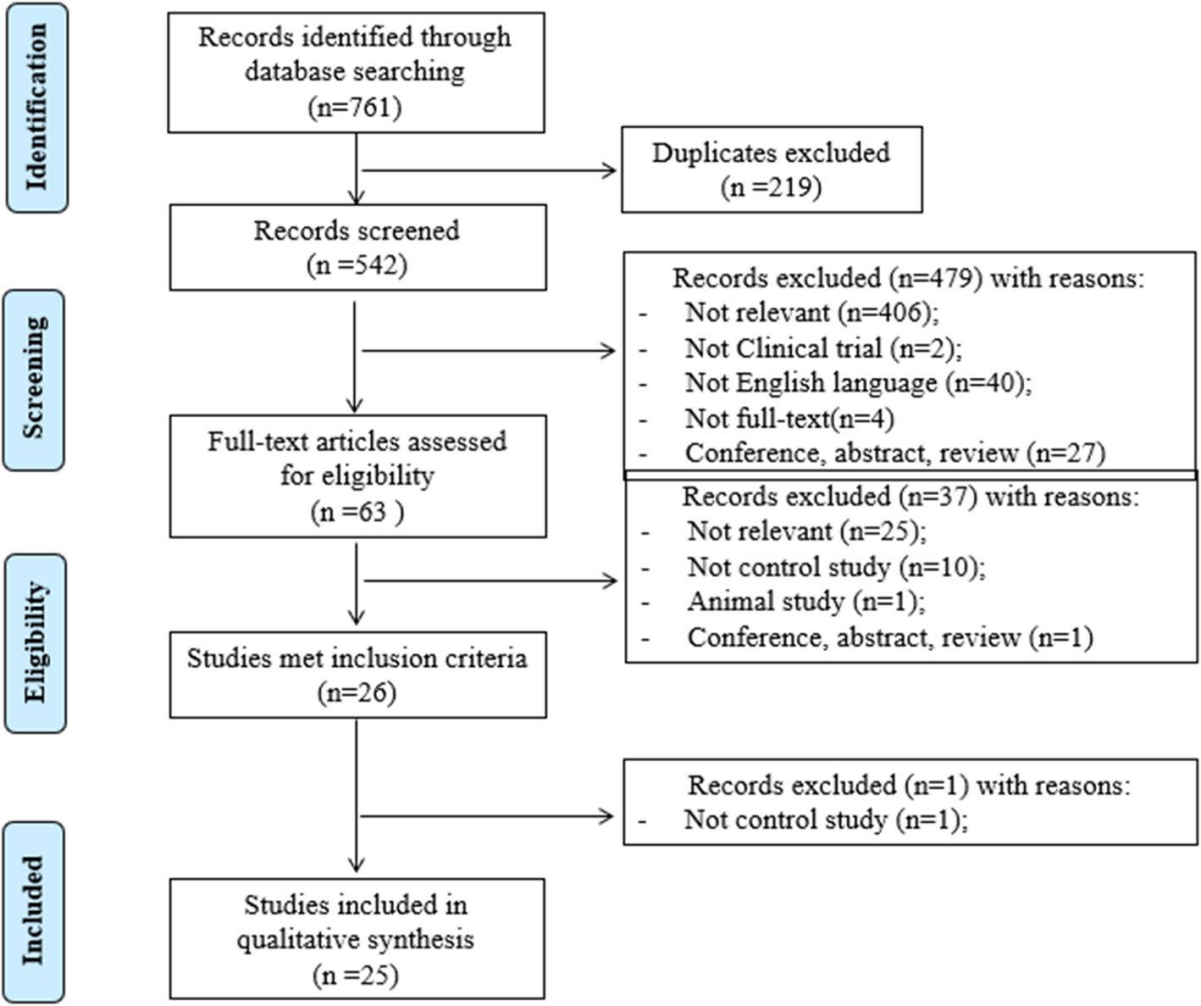
Flow diagram of the literature search and included studies

A flowchart of the literature identification and selection process is shown in Fig. 1. The initial literature search yielded 761 potentially relevant records, of which 219 were excluded. After the title and abstract screening, 63 records remained and were subjected to full-text evaluation. Ultimately, 25 studies were included in this systematic review.

### Data extraction and tabulation

The following data were extracted (Table 1): year of publication, country, author, sample size (number, sex), mean age, modalities, parameters (frequency, intensity, pulse width, wavelength, time, temperature, and depth), number of sessions, region treated, outcome measures, and findings. Four reviewers independently completed the data extraction. The outcomes of interest were extracted for the initial time point after all treatments were finished once a series of assessments were performed. This review used mean and standard deviation in the presentation of results. This review used T-test to assess certainty (or confidence).

**Table 1:** Summary of the included article

Country	Authors, year	Sample Size	Age, y	Treatment			Region Treated	Outcomes measure	Findings
				Modalities	parameters	Sessions			
Turkey	Ömer GEZGİNASLAN2020 [7]	*N* = 94 (F = 78 M = 16)	19–74	① high-energy ESWT and stretching exercises ② TENS, US, hot pack and stretching exercises	① 0.26mj/mm^2^, 2500 pulses per trigger point ② US:1 MHz, 1.5 w/cm^2^ TENS: unknown hot pack: unknown	① 7 sessions ② 10 sessions	trapezius muscles	VAS, NDI, SF36, BDI, PSQI, FACIT	Changes in the ESWT group were statistically significantly greater in these six areas. Improvement in area SF-36 was more than twice that of the control group
Germany	Márta Király2018 [15]	*N* = 61	> 18	① LLLT ② ESWT	①3 j/cm^2^ for 2 min;5000 Hz (2000 mW), 9 j/cm^2^, for 2 min ② 1000 pulses, 1.5 bar, 10 Hz, 0.25 mj/mm^2^, 15 mm treating head diamete	15 sessions	trapezius muscles	PPT, SF-36,4-grade Likert scale, NFI	Significant improvements in four areas in the two groups. ESWT group showed more obvious improvement especially in all areas of SF-36
Korea	Ki Deok Park2018 [16]	*N* = 30 (F = 27 M = 3)	19–70	① high-energy ESWT ② low-energy ESWT	① 0.210 mj/mm^2^, 1500 pulses ② 0.068 mj/mm^2^, 1500 pulses	2 sessions	upper trapezius	VAS, PPT, neck ROM, NDI	Significant improvements in four areas in the two groups. No statistically significant differences between groups in VAS and PPT Significant improvements in neck flexion and extension were observed only in the high-energy group
China	Shuo Luan2019 [17]	*N* = 62	16–60	①ESWT ②dry needling	① 0.1mj/mm^2^ ②30–50 mm deep	3 weeks	upper trapezius	VAS, PPT, NDI	Significant improvements in three areas in the two groups, but no significant differences between groups at various time points in these three areas.每组人数31
Iran	Mohammad Rahbar2020 [13]	*N* = 72	18–55	①radial ESWT and exercises ②US and exercises ③ exercises	① 2000 pulses, 60mj/m^2^,5 Hz ② 1.25 -1.5w/cm^2^ ③ unknown	① 4 sessions ② 12 sessions ③ 4 weeks	neck and upper back	VAS, PPT, NDI	Significant improvements in three areas in the first two groups than control group. ESWT group had more improvement in VAS and PPT, less improvement in NDI than US group
Turkey	Ümit Yalçın2020 [3]	*N* = 262 (F = 167 M = 95 )	20–75	①ESWT and exercises ②KT and exercises ③ exercises	① 0.056 mj/mm^2^, 1500 pulses per week ② X-shaped KT: 2 bands of 7.5 cm ③ exercises	① 3 sessions, 3 months ② 3 times, 3 months ③ months	unilateral trapeze muscle	VAS, neck ROM, NDI	Significant improvements in three areas in the first two groups than control group. ESWT group is superior to KT group in pain severity and functional recovery KT group has more significant improvement in contralateral flexion
Turkey	Ahmet Sumen2015 [6]	*N* = 45 (F = 32 M = 13)	18–65	① stretching exercises and LLLT ②stretching exercises and intramuscular electrical stimulation therapy ③ stretching exercises	① 10 Hz, 670 nm, 4 j/cm^2^, 10 min ② 80 Hz, 20 min ③ unknown, 2 times per day	① 10 sessions ② 10 days ③ 6 weeks	upper trapezius	VAS, PPT, neck ROM, NDI	Significant improvements in four areas in the first two groups than control group.LLLT group is superior to the second group only in ROM
Turkey	Umit Dundar2015 [29]	*N* = 75 (F = 75)	20–60	① high-intensity laser therapy and exercises ②placebo high-intensity laser therapy and exercises	① First phase:three subphases of 360 mj/cm^2^ (166.7 j), 410 mj/cm^2^ (166.8 j), and 510 mj/cm^2^ (166.5 j); Second phase: 610 mj/cm^2^; Third phase:three subphases of 360 mj/cm^2^ (166.7 j), 410 mj/cm^2^ (166.8 j), and 510 mj/cm^2^ (166.5 j) .15 min ②Sham-high intensity laser therapy. 15 min	15 sessions	trapezius muscles	VAS, neck ROM, NDI, SF-36	Both groups have improved in four areas.High-﻿ intensity ﻿laser group has improved more obviously in VAS, NDI and SF-36.No significant differences in neck ROM
China	Wei-Han Chang2020 [30]	*N* = 100 (F = 88 M = 12)	20–65	① acupoint LLLT ② acupoint control group ③ trigger point LLLT ④ trigger point control group	① 810 nm, 553 Hz in Shousanli,791 Hz in Houxi and 731 Hz in Waiguan ② Sham-laser therapy ③ 810 nm,583 Hz ④ Sham-laser therapy	① each acupoint 40 s ② each acupoint 40 s ③ 160 s ④ 160 s	posterior-neck and upper-back	VAS, PPT, neck ROM	Significant differences between group 3 and 4,only former had improved ipsilateral bending Significant differences between group 2 and 3, latter was possible superior for improving ipsilateral rotation. No significant difference in VAS between group 1 and 3.No significant difference in PPT among the four groups
Italy	A. Manca2014 [18]	*N* = 60 (M = 32 F = 28)	20–30	① active US ② placebo US ③ active LLLT ④ placebo LLLT ⑤ no treatment	① US: 3 MHz,1.5 W/ cm^2^ ② sham-US ③ laser wave-length 904 nm; pulse duration200ns; pulse frequency 1953 Hz; peak power 90mW; average output 30 mW; power density 22.5 mW cm2;treatment time 600 s; ④ sham-laser ⑤ no therapy	10 sessions	upper trapezius	PPT, neck ROM, NRS	Control group scored significantly less than other four groups in PPT Notably LtA scored significantly better than UsA, UsP and Control, but not than LtP in NRS No significant differences between active and placebo groups, controls scored significantly less than actives. but not than placebos in contralateral-flexion
Sri Lanka	Thusharika Dilrukshi Dissanayaka2016 [20]	*N* = 105 (F = 58 M = 47)	18–65	① TENS and standard care ② IFT and standard care ③ standard care	① 100 Hz,250 µs, 20 min ② 4000 Hz/4100HZ,20 min ③ no stimulation	8 sessions	upper trapezius	VAS, neck ROM	Improvements in the TENS group was 1.5 times that in the IFT group in VAS and neck ROM It was also larger in both TENS and IFT groups compared with control group
Turkey	Gokmen Azatcam2017 [21]	*N* = 69 (F = 48 M = 21)	18–65	① TENS and trapezius stretching exercises ② KT and trapezius stretching exercises ③ trapezius stretching exercises	① 100 ms, 60 Hz, intensity according to the paresthesia perception of the patient, 20 min ② 20 cm ③ unknown	① 10 sessions ② 4 sessions ③ 2 weeks	upper trapezius	VAS, PPT, neck ROM, NDI	TENS and KT group improved more significantly than control group in VAS.No significant difference between the groups in other areas More pronounced improvement in KT group compared to TENS group in the early period in VAS
Iran	Safoora Ebadi2021 [19]	*N* = 60 (F = 47 M = 13)	18–65	①conventional TENS ②acupuncture-like TENS ③ sham TENS	① 120 Hz, 80 µs, 30 min ② 5 Hz, 200–250 µs, 15 min ③ sham-TENS	5 sessions	upper trapezius	VAS, PPT, neck ROM,DASH	Significant improvements in VAS and DASH in favor of first two groups. Neck total lateral bending in favor of acupuncture-like TENS group compared other two groups Significant immediate improvement in all outcomes was observed only with acupuncture-like TENS
Egypt	Mary Kamal Nassif Takla2018 [22]	*N* = 70 (F = 40 M = 30)	25–45	① burst‐TENS‐combined therapy ②AMF‐combined therapy ③ sham‐combined therapy	① 100 pulses per second,2 burst per second, 43 mA, 10 min ② carrier frequency of 4,000 Hz, sweep beat frequency of 100–150 Hz, 12 mA, 10 min ③ sham‐Combined therapy, 10 min	12 sessions	upper trapezius	PPT, neck ROM	Group 1 yields a greater increase than group 2 in two areas Improvements of group 1 in PPT is about twice and in neck ROM is approximately 1.4 times that of group 2
Egypt	Mary Kamal Nassif Takla2017 [2]	*N* = 100 (F = 54 M = 46)	30–60	① PH-CT ② PH ③ US ④ sham-US	①TENS:120–200 Hz, 200 µs, US: 1 MHz continuous mode, 1.5 W/cm^2^,diclofenac sodium 10 mg gel, 10 min ② 1 MHz continuous mode, 1.5 W/cm^2^, diclofenac sodium 10 mg gel, 10 min ③ 1 MHz continuous mode, 1.5 W/cm^2^,coupling gel, 10 min ④ sham-US,coupling gel, 10 min	1 session	upper trapezius	PPT, neck ROM	Diclofenac PH-CT, PH, and US were all effective in PPT and neck ROM.PH-CT was shown to be superior over PH, and PH was superior over US in PPT.No significant difference between all the four groups in neck ROM
Turkey	Gülis Kavadar2015 [23]	*N* = 59 (F = 49 M = 10)	< 50	① conventiona-l US therapy ② placebo US therapy	① 1.5 W/cm^2^, 1 MHz ② sham-US	15 sessions	trapezius muscle	VAS, PPT, BDQ, SPMS	Significant improvements in four areas in group 1 than group 2
Turkey	Leman Ilter2013 [24]	*N* = 77 (M = 25 F = 52)	18–60	① continuous US ② pulsed US ③ sham US	① 3 MHz, 1 W/cm^2^ ② 3 MHz, 1 W/cm^2^ ③ sham-US	10 sessions	trapezius muscle	VAS, BDI, NHP, SPMS, NPDS	Significant improvements in all 5 areas in three groups. Significantly greater improvements in pain at rest in continuous US group. No statistically significant differences in the other areas
US/Thailand	Piyaraid Sakrajai2013 [36]	*N* = 31	18–65	① standard treatment and anodal tDCS; ② standard treatment and sham tDCS	① 1 mA, 20 min ② sham-tDCS, 20 min	5 days	Shoulder girdle muscles	VAS, neck ROM	Significant improvement in VAS and PPT in two groups. Significant improvement in shoulder adduction passive ROM at 1-week follow-up in group 1 than group 2
Korea	Yoon-Hee Choi2014 [37]	*N* = 21 (F = 12 M = 9)	> 18	① tDCS over M1 and trigger-point injection ② tDCS over Dorso-lateral prefrontal cortex and Trigger-point injection ③ sham tDCS and trigger-point injection	① 2 mA, 20 min ② 2 mA, 20 min ③ Sham-tDCS, 20 min	5 days	Shoulder girdle muscle	VAS, PPT, SF-MPQ	Mean VAS values were decreased in all three groups, significant change only in group 2.No significant differences among the three groups in PPT and SF-MPQ
China	Jingyun Xu2021 [38]	*N* = 68 (F = 68)	18–70	① biofeedback and electrical stimulation, self-myofascial release ② self-myofascial release	① first program:50–280 Hz,50 µs second program: 1–10 Hz, 200 µs third program:1–2 Hz, 300 µs ② no stimulation	14 sessions	pelvic muscles	NRS, PGI-I, sEMG, Degree of activation of MTrPs	Improvements in four areas in two groups. Greater change in group 1 in NRS and degree of activation of MTrPs, sEMG levels of pretest resting baseline and posttest resting baseline. No between-group differences in sEMG levels of quick flicks and endurance contraction Group 1 improved PGI-I at 4-week post-intervention but not at 12-week post-intervention
Iran	Fariba Eslamian2020 [8]	*N* = 50 (F = 39 M = 11)	25–55	①acupuncture and concomitant electrical stimulation ②biofeedback	① ES:200 µs,100 Hz,10 -45 mA, 30 min ② Parameters were unknown, 30 min	6 sessions	neck and upper back	VAS, PPT, neck ROM, NDI	Significant improvements in two groups, intergroup differences showed priority of acupuncture in all four areas. Change in group 1 was almost 3 times that in group 2 in NDI
United Arab Emirates	Ibrahim M. Moustafa2018 [39]	*N* = 120 (F = 44 M = 76)	Unknown	① denneroll cervical traction ② placebo treatment	unknown	30 sessions	scapular retractors, deep cervical flexors, and neck extensors	PPT, neck ROM, NDI, NRS	Equal improvements for both groups in NDI and NRS. Significant differences between groups favoring group 1 for PPT, sagittal alignment variables and all measures of neck ROM at 10 weeks. Significantly difference between groups favoring group 1 at 1-year follow up all variables
China	Yen-Ting Lai2017 [40]	*N* = 117 (F = 78 M = 39)	> 18	① FIR patch ② placebo treatment	① 0.038 w/cm^2^,24 h ② no stimulation	1 session	trapezius muscle	VAS, PPT, MPT	Significant improvement in VAS in two groups. Only FIR group had change in PPT and MPT
Spain	Mireia Yeste-Fabregat2021 [41]	*N* = 32	16–39	① TT ② sham-TT	① 500 MHz, 40% of the maximum intensity, 25 min ② sham-TT, 25 min	1 session	gastrocnemius muscle	VAS, lunge test, absolute temperature	Greater increase in absolute temperature in group 1 than group 2 No difference between the groups in lunge test
Korea	Sang Hee Im2013 [9]	*N* = 41 (F = 13 M = 28)	Unknown	① whirlpool bath ② conventional hydrocollator pack	① 32—36℃, 30 min ② 74.5℃, 30 min	6 sessions	upper trapezius	VAS, BDI, QOL	Greater improvements in VAS and BDI of group 1 than that of group 2 No significant difference between two groups in QOL

*AMF* Amplitude modulated frequency, *BDI* Beck Depression Inventory, *BDQ* Beck Depression Questionnaire, *DASH* Disabilities of the Arm, Shoulder, and Hand, *ESWT* Extracorporeal shock wave therapy, *F* Female, *FACIT* Functional Assessment of Chronic Illness Therapy, *FIR* Far-infrared ray, *IFT* Interferential therapy, *KT* Kinesiological taping, *LLLT* Low-level laser therapy, *M* Male, *MPT* Maximal pain tolerance, *NDI* Neck Disability Index, *NFI* Neck Functionality Impairment, *NHP* Nottingham Health Profile, *NPDS* Neck Pain and Disability Scale, *NRS* Numerical rating scale, *PH* phonophoresis, *PH-CT* Phonophoresis combined therapy, *PGI-I* Patient Global Impression of Improvement, *PPT* Pain pressure threshold, *PSQI* Pittsburgh Sleep Quality Index, *QOL* Quality of life, *ROM* Range of motion, *SF-36* Medical Outcomes Study 36-item Short Form health survey, *sEMG* Surface electromyography, *SF-MPQ* Short Form McGill Pain Questionnaire, *SPMS* Severity of palpable muscle spasm (five-step scale), *tDCS* Transcranial direct-current stimulation, *TENS* transcutaneous electrical nerve stimulation, *TT* Transfer energy capacitive and resistive therapy, *US* Ultrasound, *VAS* Visual analogue scale

### Risk of bias

Three reviewers independently assessed the risk of bias of the included studies without author or journal blinding. Risk of bias was determined using parameters outlined in the Cochrane Handbook for Systematic Reviews. Selection, performance, detection, attrition, reporting, and others were assessed and rated as low, unclear, or high risk of bias. A low risk of bias was assigned if the authors described their methodology toward mitigating the item of interest; an unclear risk of bias was assigned if the authors did not discuss the item; and a high risk of bias was assigned if the authors reported a limitation of the item of interest or the reviewer saw a way the bias could affect the results. For “performance bias,” it is difficult to implement blind intervention measures for subjects and researchers because of the particularity of therapeutic physical modalities. Therefore, if the evaluator was unaffected by the unblinding or imperfect blinding method when judging the outcome, a low-risk rating was given. Table 2 shows the details of each included trial.

**Table 2:** Risk of bias of included study

Study	selection bias		performance bias	detection bias	attrition bias	reporting bias	other bias	final judgment
	random sequence generation	allocation hidden	Blinding of patients and trial staff	Blinding of outcome assessors	Incomplete result data	selective reporting		
Ömer GEZGİNASLAN2020 [7]	-	-	?	-	-	-	-	certain risk
Márta Király 2018 [15]	-	-	-	-	-	-	-	low risk
Ki Deok Park 2018 [16]	-	+	-	-	-	-	?	high risk
Shuo Luan2019 [17]	-	-	-	-	-	-	?	certain risk
Mohammad Rahbar2020 [13]	-	-	-	-	-	-	-	low risk
Ümit Yalçın 2020 [3]	?	?	?	?	-	-	?	certain risk
Ahmet Sumen 2015 [6]	-	?	?	-	-	-	-	certain risk
Umit Dundar 2015 [29]	-	?	-	-	-	-	-	certain risk
Wei-Han Chang2020 [30]	-	-	-	-	-	-	?	certain risk
A.Manca2014 [18]	-	-	-	-	-	-	?	certain risk
Thusharika Dilrukshi Dissanayaka 2016 [20]	-	?	-	-	-	-	?	certain risk
Gokmen Azatcama 2017 [20]	-	?	?	-	-	-	-	certain risk
SafooraEbadi2021 [19]	-	-	-	-	-	-	-	low risk
Mary Kamal Nassif Takla2018 [22]	-	-	-	-	-	-	-	low risk
Mary Kamal Nassif Takla2018 [2]	-	-	-	-	-	-	?	certain risk
GülisKavaDaR2015 [23]	-	-	?	-	-	-	?	certain risk
Leman Ilter 2013 [24]	-	-	-	-	-	-	-	low risk
PiyaraidSakrajai2013 [36]	?	?	?	?	-	-	?	certain risk
Yoon-Hee Choi, MD 2014 [37]	-	-	-	?	-	-	-	low risk
Jingyun Xu 2021 [38]	-	-	-	?	-	-	?	certain risk
Fariba Eslamian 2020 [8]	-	-	-	-	+	-	?	high risk
Ibrahim M. Moustafa 2018 [39]	-	-	+	?	-	-	?	high risk
Yen-Ting Laia 2017 [40]	-	-	-	-	+	-	?	high risk
Mireia Yeste-Fabregat 2021 [41]	-	-	-	-	-	-	?	certain risk
Sang HeeIm 2013 [9]	?	?	?	?	?	-	?	certain risk

*Note*: + , low risk of bias; -, high risk of bias; ?, unclear risk of bias

## Outcomes of interest

### Extracorporeal shock wave therapy

Six studies used ESWT [3, 7, 13, 15–17]. Three of them used only low-energy ESWT, [3, 13, 17] two used high-energy ESWT, [7, 15] and one used both [16]. In six studies, the VAS score and NDI of the ESWT group were significantly decreased versus baseline. Park et al. showed statistically significant improvement in neck flexion and extension in the high-energy ESWT group only [16].

ESWT effectively treats MPS. Five articles have studied whether ESWT is superior to other treatments. Márta et al. and Ömer et al. reported that ESWT had more advantages than low level laser therapy(LLLT) and the combination of hot pack, TENS and US in area SF-36 [7, 15]. Ümit et al. and Rahbar et al. also indicated that ESWT was superior to kinesiological taping (KT) and US in relieving pain severity [3, 13]. Apart from the above four studies, only one study reported that ESWT and acupuncture both achieved significant improvements in VAS, PPT and NDI areas, but no significant difference between them [17].

Some researchers compared the therapeutic effects of ESWT at different intensities. Park et al.’s study found that high-energy EWST is superior to low-energy ESWT in neck flexion and neck function improvement [16]. However, the results are only low effect quantities (0.47, 0.41). Sugawara et al. reported that radical ESWT had the best therapeutic effect on the MPS patients with severe pain (VAS score > 70 mm) at high frequency (> 15 Hz) [27]. Merzgnaslan et al. confirmed that high-energy ESWT is more effective than the combination of hot pack, TENS, and US, especially according to the SF-36 [7].

The treatment parameters of ESWT included: treatment intensity, 0.056–0.25 mJ/mm^2^; 1000–2500 pulses; and 3–15 treatment sessions. The mechanism of ESWT in relieving MPS pain may include aiding blood vessel reconstruction, increasing blood perfusion and tissue oxygen saturation, changing the pain signal in ischaemic tissue caused by calcium inflow [3, 7, 12, 13, 15–17, 28] by causing transient dysfunction of nerve excitability at the neuromuscular junction through selective partial denervation (degeneration of the acetylcholine receptor in free nerve endings), [7, 16, 17] stimulating fibroblast production within connective tissues such as tendon ligaments and fascia, stimulating the release of local growth factors and promoting the repair of damaged tissues, [3, 15, 27] and reducing musculoskeletal pain by reducing substance P (Neurokinin P) production in the dorsal root ganglia [7].

### Laser therapy

Four studies investigated the effect of laser irradiation on MPS [6, 18, 29, 30]; all included sham laser as a control. One study included 1 treatment session, while the rest included 10–15 treatment sessions [30]. Chang et al. reported that the improved changes in VAS and ROM of cervical ipsilateral flexion and rotation caused by a single LLLT treatment applied to myofascial trigger points [30]. The remaining three studies reported that pain in the LLLT treatment group was significantly reduced, [6, 18, 29] while two trials reported significant intergroup differences [6, 29]. One study evaluated the effectiveness of high-intensity laser therapy and sham laser therapy in female patients with chronic MPS of the trapezius muscle [29]. Significant post-treatment intra- and intergroup differences were noted in pain, NDI, and SF-36 scores in high-intensity laser group. LLLT was used in other three studies, and significant within group differences were observed in PPT and pain [6, 18, 30]. At the same time, LLLT was superior to US and intramuscular electrical stimulation, but no significant difference was noted in pain score or PPT when LLLT was used on different points (acupoints and MTrPs).

The treatment parameters of the laser therapy were as follows: frequency, 553 ~ 5000 Hz; wavelength, 670 ~ 904 nm; intensity, 0.025 ~ 9 J/cm^2^; time, 40 s ~ 10 min for each point; and treatment session, 1–15 times. The laser induces photochemical and photothermal effects on the surface or deep tissues, thus increasing local microcirculation to increase the oxygen supply, expedite the elimination of local metabolites, and relieve pain [6, 29]. Improving β-endorphin precursor mRNA expression achieves an analgesic effect in inflamed peripheral tissues [31]. Animal studies showed that LLLT at 4.5 J/cm^2^ diminished tumour necrosis factor-α levels in the tissue and cyclooxygenase-2 (COX-2) expression in the muscle, while LLLT at 27 J/cm^2^ increased the β-endorphin precursor level in the serum, dorsal root ganglia, and muscle [32]. It also reduced COX-2 mRNA and c-Fos expressions in the central nervous system (CNS), reduced hyperalgesia, and relieved pain [33, 34].

### Transcutaneous electrical nerve stimulation

Four studies explored the effect of TENS on MPS of the superior trapezius muscle [19–22]. All studies included a control group. The neck ROM of the four studies was significantly improved; three reported significantly improved intra- and intergroup pain, [19–21] the intragroup PPT of three studies decreased significantly [19, 21, 22]. Electrical stimulation is effective at different frequencies, but low-frequency, high-intensity TENS more effectively affects pain sensitivity and ROM of MPS patients than other electrical stimulation treatments. Ebadi et al. compared the therapeutic effects of acupuncture-like TENS (AL-TENS), characterised by low frequency and high intensity, with conventional TENS (C-TENS), characterised by high frequency and low intensity, and reported that both can relieve pain and pressure thresholds and improve functional performance for up to 3 months; only AL-TENS improved neck lateral flexion ROM [19]. Klamakn et al. studied the therapeutic effects of low-frequency, high-intensity burst TENS and medium-frequency, low-intensity amplitude-modulated frequency (AMF) combined with US and confirmed their efficacy for decreasing PPT and increasing neck lateral flexion ROM [22]. The PPT and ROM in the burst TENS group were approximately twice and 1.4 times those of the AMF group, respectively. The difference were high effect quantities(0.89, 0.86, 0.89). Dissanayaka et al. also reported that the patients with MPS has experienced greater improvement of pain and physical function in the TENS group than in the interferential therapy (IFT) group [20]. Although the similar mechanisms exist in TENS and IFT, one possible reason for the better effect of the former was that its electrodes were placed on MTrPs, whereas those of the latter were placed around MTrPs, which might reduce the current density transmitted to MTrPs. Furthermore, Ahmed et al. indicated that a single treatment of TENS with longer than 15 min would be more effective than those with a short treatment time [1]. Apart from the above three studies, only one study reported no advantage of TENS over kinesiological taping in the treatment of MPS [21].

The summarised treatment parameters of TENS were as follows: frequency, 5 ~ 100 Hz; pulse width, 80 µs to 100 ms; treatment duration, 10–30 min; and number of treatment sessions, 5 ~ 12. According to International Association for the Study of Pain classification, TENS is divided into C-TENS and AL-TENS. C-TENS stimulates large, low-threshold afferents to inhibit second-order nociceptive transmission cells for a few minutes, whereas AL-TENS activates high-threshold afferents, suppressing central nociceptive transmission for at least 1 h [19, 20, 22]. Studies confirmed the mechanism of central analgesia; namely, TENS significantly decreased substance P overexpression, enhanced MOR expression in the parabrachial nucleus, and elevated c-Fos expression in the rostral ventromedial medulla [21, 35].

### Ultrasound

Three studies explored US efficacy for treating MPS; all included a sham US group [18, 23, 24]. All studies reported that low-intensity US could significantly reduce the pain of MPS, and two reported significant intergroup differences [23, 24]. Kavadar et al. and Manca et al. reported significant intragroup differences in pain pressure threshold [18, 23]. Ilter et al. compared the therapeutic effects of continuous and pulsed US and found the superior effect of continuous one on the reduction of resting pain [24].

The treatment parameters of US were as follows: frequency, 1–3 MHz; intensity, 1–1.5 W/cm^2^; and number of treatment sessions, 10–15. US can relieve MPS pain by increasing the permeability of blood vessels and cell membranes and promoting angiogenesis and microcirculation, thus promoting muscle relaxation and increasing connective tissue extensibility [2, 24]. Furthermore, the analgesic effect of US on MPS may be attributed to the modulation mechanism of central nervous pathway. Nitric oxide (NO) and NO synthase (NOS) play roles in promoting central sensitisation mechanisms and inflammatory hyperalgesia [23]. With the influence of persistent nociceptive input, the number of neuronal nitric oxide synthase like neurons(nNOS-LI) neurons increases in the dorsal horns of spinal cord, resulting in increased NO and substance P synthesis. This process could be inhibited by US, with the obvious decrease of nNOS-LI in the dorsal horns and thus pain relief.

The study also clarified the therapeutic mechanism of phonophoresis and US combined with TENS. Phonophoresis has an US-like effect that also increases stratum corneum permeability based on the thermal effect of US, promotes drug diffusion on the skin’s surface, and increases drug absorption by the skin and deep tissues [2, 23, 24]. US decreases the resting potential of the nerve cell membrane, resulting in increased permeability to sodium and calcium ions and bringing the nerve membrane closer to the depolarisation point, but the nerve fails to fire. The simultaneous application of TENS through partially depolarised nerves induces further depolarisation, inducing action potentials. The application of TENS through partially depolarised nerves induces further depolarisation, inducing action potentials [2, 22].

### Transcranial direct-current stimulation

Two studies investigated the effectiveness of tDCS at relieving pain in the patients with MPS [36, 37]. In both studies, pain in the tDCS stimulation groups was significantly decreased, but it did not change significantly compared to sham tDCS. At the same time, tDCS stimulation of the dorsolateral prefrontal cortex more effectively relieved pain than that of the M1 cortex [37].

The summarised tDCS treatment parameters were: intensity, 1–2 mA; treatment duration, 20 min; and 5 treatment sessions. The therapeutic mechanism of tDCS in MPS may be associated with reversal of the central pain pathway by regulating cortical plasticity [37]. One study proposed that patients with chronic pain may have intrinsic cortical inhibition defects [36]. Moreover, tDCS induces a weak constant current that changes the resting membrane potential and increases the overall discharge activity in the cortex area immediately below the anode electrode. Therefore, tDCS may promote the activities of brain regions that suppress pain signals. Active tDCS may also increase synaptic transmission through the *N-*methyl-d-aspartate (NMDA) receptors [36].

### Biofeedback

Two studies investigated the effect of biofeedback on MPS; however, neither included a sham treatment group as a control [8, 38]. Both reported that the biofeedback and its combination with other treatments could significantly reduce the pain, improve the muscle function, and increase the NDI and neck ROM in the patients with neck and pelvic floor MPS [8, 38].

The summarised parameters of biofeedback were as follows: treatment time, 20 ~ 30 min; and number of treatment sessions, 6 ~ 14. Biofeedback therapy has the advantage of reducing the involuntary muscle contraction, improve the uncoordinated muscle movement, and enhance the muscle strength to relieve fatigue and improve joint mobility.

### Traction

Traction for MPS was used in one study. Researchers applied neck traction in patients with chronic MPS for 2 ~ 3 min and increased it by 1 min per session until it reached 20 min [39]. After treatment, significant intergroup differences were noted in the PPT and neck ROM. Immediately post-treatment and after 1-year follow-up, significant differences in NRS, NDI, PPT, and neck ROM were noted.

This study also reported that long-term abnormal posture can cause MPS. Changes in the sagittal-plane arrangement of the vertebral bodies may cause abnormal stress and strain, leading to early and accelerated muscle, ligament, bone structure, and nerve degeneration, thus causing chronic MPS. Traction can restore normal vertebral alignment, positively impacting pain, function, and mobility.

### Far-infrared ray

Lai et al. studied the effect of FIR and placebo patches on MPS, and the intervention group received 24-h treatment at an intensity of 0.038 w/cm^2^ [40]. The VAS scores were similar between groups; however, only the PPT and maximal pain tolerance of the FIR intervention group decreased significantly post-treatment. FIR leads to vasodilatation, improves metabolism, reduces blood and body fluid viscosity, and increases pain threshold through thermal effects. Therefore, its ability to relieve MPS pain and tissue adhesion is essential.

### Transfer energy capacitive and resistive therapy

One study focused on the immediate effects of TECAR therapy on skin temperature, ankle joint mobility, and hyperalgesia in MPS patients [41]. Patients with gastrocnemius MPS were treated; of them, 15 in the intervention group were given TECAR therapy at a frequency of 500 MHz, intensity of 40%, and treatment duration of 25 min, while 17 in the control group were treated with sham TECAR therapy. The absolute body temperature was significantly higher in the intervention versus control group. However, there were no significant intergroup differences in lunge test or VAS score. Therefore, the effect of TECAR therapy for MPS requires further study.

TECAR therapy can reportedly reduce pain in patients with osteoarthritis [42]. The main mechanism of TECAR therapy is that the interaction of radiofrequency currents with biological structures increases the endogenous temperature, and the generated thermal effect can alleviate pain by promoting the vasodilation of tissues affected by pain mediators such as bradykinin, serotonin, and prostaglandins while reducing muscle spasm, accelerating cell metabolism, and increasing soft-tissue extensibility.

### Whirlpool bath

One study compared the efficacy of a whirlpool bath and hot pack on MPS. The intervention group was treated with 6 sessions of a 32 ~ 36 °C whirlpool bath for 30 min [9]. The control group received a standard hot pack for 30 min. Pain and anxiety was improved significantly in the intervention group; however, no significant intergroup difference in QOL improvement was noted. Warm and hot water whirlpool baths have thermal and mechanical effects that can close the pain gate and relieve pain through gentle mechanical stimulation [43].

## Discussion

Therapeutic physical modalities are widely administered to treat MPS in clinical settings. This review examined the RCTs of therapeutic physical modalities in the treatment of MPS to confirm its safety and effectiveness, mechanism, optimal treatment protocol and parameters. The studies included in this review involve ten kinds of therapeutic physical modalities. Owing to the heterogeneity of the included studies, a planned meta-analysis was impossible. The results of these studies all indicated that therapeutic physical modalities play a significant role in promoting pain, joint mobility, psychological state, and QOL of patients with MPS with no side effects. From the perspective of pain reduction, the TECAR therapy has no positive evidence for MPS-induced pain. The anti-nociceptive effect of tDCS intervention has been demonstrated, which was associated with the area in the dorsolateral prefrontal cortex [37]. The continuous US plays a more positive role in alleviating the resting pain in the patients with MPS than the pulsed US [24]. Furthermore, the high-frequency ESWT with frequency of more than 15 Hz has a better therapeutic effect on the MPS patients with severe pain (VAS score > 70 mm) [27]. From the perspective of physical function, ESWT has been found significantly to reduce the NDI score, in which high-energy ESWT was more effective than low-energy ESWT for the patients with moderate and high pain intensity (VAS Score > 40 mm) [16]. Among the electrical stimulation treatments, low-frequency, high-intensity TENS provides greater improvement in the neck ROM, [19, 20, 22] especially at burst‐TENS group.

At the same time, the synergistic effect of therapeutic physical modalities has been shown in a large number of included studies. The combined therapeutic physical modalities therapy had a better therapeutic effect than the single therapy. Four studies showed that TENS, EWST, and LLLT combined with exercise were more effective than exercise alone [3, 6, 13, 21]. Jingyun et al. showed that the combined therapy of biofeedback, electrical stimulation, and self-myofascial release could significantly reduce the pelvic myofascial pain [38]. Mary et al. also pointed out that the combined therapy of phonophoresis and TENS could obviously reduce the sensitivity of active MTrPs than US or phonophoresis alone [2]. The therapeutic effect of tDCS plus MTrPs injection has been found to be better than that of MTrPs injection alone [37]. However, there are some problems with existing trials, such as disunity of the combined therapy and the inconsistency of treatment parameters such as frequency, intensity, and duration. Therefore, the optimized intervention combination and specific parameters of therapeutic physical modalities require further exploration.

The etiological mechanism of MPS is mainly the symptoms of chemical pain, swelling and skin temperature rise caused by inflammatory reactions such as ischemia, swelling and accumulation of inflammatory mediators caused by tissue injury [1–5, 10–13]. Then, the involved pain caused by nerve sensitization, muscle compensatory spasm caused by energy crisis, tissue sclerosis caused by connective tissue hyperplasia, and the clinical symptoms characterized by high irritation nodules in the muscle tension zone, pain and limited ROM were formed [1–5, 10–13]. Therefore, improving blood circulation, promoting the metabolism of inflammatory mediators and eliminating swelling as soon as possible in the acute stage of inflammation is the key to avoid subsequent nerve sensitization and energy crisis. Chronic stage can be further treated by regulating nerve conduction pathway, correcting posture and improving muscle function. At the same time, the treatment plan should be set according to the changes of the pathological process of the disease. Different kinds of therapeutic physical modalities have different mechanisms in treating MPS, but they all achieve therapeutic effects by increasing blood perfusion and blood oxygen supply in ischemic tissues, reducing hyperalgesia in peripheral and central nerves, and improving involuntary muscle contraction [3, 7, 13, 15–24].

Because of the obvious heterogeneity of the included studies, we could not draw a set of standardised treatment prescriptions; rather, we could only summarise some commonalities between clinical medical staff and researchers’ references. Low-frequency, high-intensity TENS with a frequency of 2–4 Hz for a duration longer than 15 min and tDCS are used to reduce peripheral and central nervous hyperalgesia [19–22, 36, 37]. Using continuous US to relieve resting pain [18, 23, 24]. Traction is used to improve the influence of abnormal stress and strain on muscles and other tissues [39]. Using biofeedback to improve involuntary muscle contraction [8, 38]. The mechanical stress effect and cavitation effect of ESWT make blood vessels and soft tissue cells undergo the process of collapse and regrowth [3, 7, 13, 15–17]. Therefore, the symptoms of patients at the initial stage of treatment will be aggravated due to the new inflammatory reaction, and it is suitable for patients with chronic inflammatory phase and lingering. Wax therapy, Laser, hot pack, warm-heat diathermy, warm or hot water whirlpool bath etc. are used to improve blood perfusion and blood oxygen supply of ischemic tissue, relieve muscle spasm, loosen tissue viscosity and improve joint mobility in chronic stage. On the basis of the included studies, therapeutic physical modalities intervention is recommended to combine with exercise, manipulation, and MTrPs injection. Although the clinical mechanisms of various therapeutic physical modalities treatments for MPS have been clarified, only ESWT, TENS, and LLLT have been proven in animal studies, while basic research on other treatments is still lacking.

The included studies in this review have been analysed and its clinical trial design requires further improvement. First of all, different treatments and parameters should be set for patients at different stages of tissue repair. However, the existing studies rarely screen patients at the same stage to observe the therapeutic effect, and do not adopt different treatment parameters for patients at different stages. Therefore, the conclusion of the article should be carefully adopted in clinical practice, and a more accurate experimental design is also needed. In the second place, most studies paid insufficient attention to whether the therapeutic effect can be maintained long term. Only 14 of the 25 RCTs examined the long-term effects (including follow-up periods of 1 week, 2 weeks, 1 month, 3 months, and 1 year). The rest of the clinical studies only observed the immediate treatment effect. In contrast, Sakrajai et al. reported that the significant difference in pain between groups disappeared in the second week of follow-up [36]. The reason for the relatively short duration of treatment effect may be that it is affected by the placebo effect, which is not the best treatment prescription, and the longest expected benefit duration of tDCS (without intensive treatment) may be 1–2 weeks. Therefore, it is necessary to observe long-term therapeutic effects. Thirdly, the influence of therapeutic physical modalities on the psychological state, sleep, and QOL of patients with MPS was less involved in these RCT studies. The main symptoms of MPS are pain and limited joint mobility. Patients with MPS often have psychological problems such as anxiety, depression, and disordered sleep, which significantly reduces the QOL [44]. However, only 6 studies in this review focused on QOL, 4 focused on psychological state, and 1 focused on sleep. It is necessary to objectively evaluate the MPS patients’ psychology, sleep, and other aspects after a long-term duration of therapeutic physical modalities intervention. Finally, the therapeutic effect of therapeutic physical modalities may be affected by the patients’ subjective feedback, psychological factors, drugs, accuracy of therapeutic operation, or skin sensitivity. Moreover, the PPT, VAS, NRS, and other assessments used in a large number of studies to assess pain mainly depend on the patients’ subjective feedback and sensitivity [18]. Manca et al. reported no advantage of the intervention over placebo, so the therapeutic effect might be influenced by psychological factors [18, 45]. Some patients in the Xu et al. study were taking analgesics and muscle relaxants simultaneously, which may have also affected the research results [38]. Sakrajai et al. reported that tDCS therapeutic electrode accuracy affects the area outside the M1 cortex and subsequently affects the therapeutic effect [36]. Lai et al. reported that the pre-treatment evaluation showed that the PPT was positively correlated with the maximum pain tolerance, so the worse the skin sensitivity and the higher the age, the higher the PPT values and maximum pain tolerance [40]. However, studies are lacking on the effects of therapeutic physical modalities on MPS in the patients with different skin sensitivities and ages. Future research is warranted to different pathological stages and conduct a long-term follow-up to clarify the long-term curative effect of therapeutic physical modalities, and its relevant impact factors.

## Conclusion

The present articles showed that therapeutic physical modalities effectively improve the pain, PPT, ROM and QOL of patient with MPS. This review summarized the types, methods and parameters of therapeutic physical modalities for MPS, which provides evidence for clinical application. However, there are a series of problems in existing research, such as inconsistent treatment prescriptions and parameters and insufficient sample sizes. At the same time, research on magnetic, wax, and other therapeutic physical modalities treatments for MPS is lacking. Future clinical research should focus on the optimized treatment parameters of therapeutic physical modalities in different inflammatory stages and the combination therapy to construct the significant treatment prescription for MPS clinical reference. It is also necessary to pay attention to the psychology, sleep, skin sensitivity, long-term efficacy, and other factors of MPS and expand the sample size for further research. Finally, basic research is needed to clarify the mechanisms of different treatments of therapeutic physical modalities.

## Data Availability

The datasets used and/or analysed during the current study available from the corresponding author on reasonable request.

## Abbreviations

AMF: Amplitude modulated frequency
BDI: Beck Depression Inventory
BDQ: Beck Depression Questionnaire
DASH: Disabilities of the Arm, Shoulder, and Hand
ESWT: Extracorporeal shock wave therapy
F: Female
FACIT: Functional Assessment of Chronic Illness Therapy
FIR: Far-infrared ray
IFT: Interferential therapy
KT: Kinesiological taping
LLLT: Low-level laser therapy
M: Male
MPT: Maximal pain tolerance
NDI: Neck Disability Index
NFI: Neck Functionality Impairment
NHP: Nottingham Health Profile
NPDS: Neck Pain and Disability Scale
NRS: Numerical rating scale
PH: Phonophoresis
PH-CT: Phonophoresis combined therapy
PGI-I: Patient Global Impression of Improvement
PPT: Pain pressure threshold
PSQI: Pittsburgh Sleep Quality Index
QOL: Quality of life
ROM: Range of motion
SF-36: Medical Outcomes Study 36-item Short Form health survey
sEMG: Surface electromyography
SF-MPQ: Short Form McGill Pain Questionnaire
SPMS: Severity of palpable muscle spasm (five-step scale)
tDCS: Transcranial direct-current stimulation
TENS: Transcutaneous electrical nerve stimulation
TT: Transfer energy capacitive and resistive therapy
US: Ultrasound
VAS: Visual analogue scale
MPS: Myofascial pain syndrome
MTrPs: Myofascial trigger points
PubMed: Performed of Medline
MeSH: Medical subject heading terms
RCTs: Randomised controlled trials
COX-2: Cyclooxygenase-2
CNS: Central nervous system
AL-TENS: Acupuncture-like TENS
C-TENS: Conventional TENS
NO: Nitric oxide
NOS: NO synthase
nNOS-LI: Neuronal nitric oxide synthase like neurons
NMDA: *N-*Methyl-d-aspartate
